# Tris(*cis*-2-hy­droxy­cyclo­hexane-1,3,5-tri­aminium) hydrogen sulfate octa­chloride dihydrate

**DOI:** 10.1107/S1600536812022374

**Published:** 2012-05-26

**Authors:** Christian Neis, Günter J. Merten, Kaspar Hegetschweiler

**Affiliations:** a Fachrichtung Chemie, Universität des Saarlandes, Postfach 151150, D-66041 Saarbrücken, Germany

## Abstract

The 2-hy­droxy­cyclo­hexane-1,3,5-triaminium (= H_3_ *L* ^3+^) cation of the title compound, 3C_6_H_18_N_3_O^3+^·8Cl^−^·HSO_4_^−^·2H_2_O, exhibits a cyclo­hexane chair with three equatorial ammonium groups and one axial hy­droxy group in an all-*cis* configuration. The hydrogen sulfate anion and two water mol­ecules lie on or in proximity to a threefold axis and are disordered. The crystal structure features N—H⋯Cl and O—H⋯Cl hydrogen bonds. Three *C*_3_-symmetric motifs can be identified in the structure: (i) Two chloride ions (on the *C*_3_-axis) together with three H_3_ *L* ^3+^ cations constitute an [(H_3_ *L*)_3_Cl_2_]^7+^ cage. (ii) The lipophilic C_6_H_6_-sides of three H_3_ *L* ^3+^ cations, which are oriented directly towards the *C*_3_-axis, generate a lipophilic void. The void is filled with the disordered water mol­ecules and with the disordered part of the hydrogen sulfate ion. The hydrogen atoms of these disordered moieties were not located. (iii) Three H_3_ *L* ^3+^ cations together with one HSO_4_^−^ and three Cl^−^ counter-ions form an [(HSO_4_)(H_3_ *L*)_3_Cl_3_]^5+^ cage. Looking along the *C*_3_-axis, these three motifs are arranged in the order (cage 1)⋯(lipophilic void)⋯(cage 2). The crystal studied was found to be a racemic twin.

## Related literature

The synthesis of a sulfate salt of H_3_ *L* ^3+^ as well as metal complex formation of *L* has been reported by Merten *et al.* (2012 ▶). For the synthesis of a diastereomeric form of *L*, see: Castellanos *et al.* (1980 ▶). The hydrogen-bonding ability of axial *versus* equatorial hy­droxy groups is discussed by Bonnet *et al.* (2005 ▶), and further examples in related structures are provided by Neis, Merten & Hegetschweiler (2012 ▶) and Neis, Merten, Altenhofer & Hegetschweiler (2012 ▶). Puckering parameters have been calculated according to Cremer & Pople (1975 ▶). For the treatment of hydrogen atoms in *SHELXL*, see: Müller *et al.* (2006 ▶).
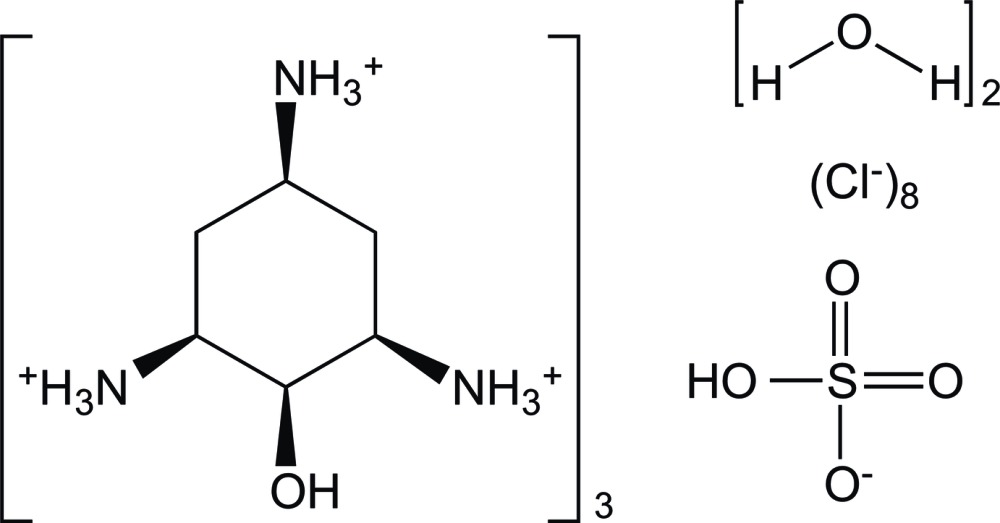


## Experimental

### 

#### Crystal data

3C_6_H_18_N_3_O^3+^·8Cl^−^·HSO_4_^−^·2H_2_O
*M* *_r_* = 861.40
Trigonal, 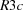

*a* = 12.6549 (18) Å
*c* = 43.616 (9) Å
*V* = 6049.2 (17) Å^3^
*Z* = 6
Mo *K*α radiation
μ = 0.66 mm^−1^
*T* = 200 K
0.48 × 0.40 × 0.32 mm

#### Data collection

Stoe IPDS image plate diffractometer
14259 measured reflections
2518 independent reflections
2442 reflections with *I* > 2σ(*I*)
*R*_int_ = 0.075

#### Refinement

*R*[*F* ^2^ > 2σ(*F* ^2^)] = 0.037
*wR*(*F* ^2^) = 0.101
*S* = 1.07
2518 reflections
169 parameters
11 restraints
H atoms treated by a mixture of independent and constrained refinement
Δρ_max_ = 0.75 e Å^−3^
Δρ_min_ = −0.32 e Å^−3^
Absolute structure: Flack (1983 ▶), 1255 Friedel pairs
Flack parameter: 0.41 (7)

### 

Data collection: Stoe *IPDS Software* (Stoe & Cie, 1997 ▶); cell refinement: Stoe *IPDS Software*; data reduction: Stoe *IPDS Software*; program(s) used to solve structure: *SHELXS97* (Sheldrick, 2008 ▶); program(s) used to refine structure: *SHELXL97* (Sheldrick, 2008 ▶); molecular graphics: *DIAMOND* (Brandenburg, 2012 ▶); software used to prepare material for publication: *SHELXL97* and *PLATON* (Spek, 2009 ▶).

## Supplementary Material

Crystal structure: contains datablock(s) global, I. DOI: 10.1107/S1600536812022374/nk2161sup1.cif

Structure factors: contains datablock(s) I. DOI: 10.1107/S1600536812022374/nk2161Isup2.hkl

Additional supplementary materials:  crystallographic information; 3D view; checkCIF report

## Figures and Tables

**Table 1:** Hydrogen-bond geometry (Å, °)

*D*—H⋯*A*	*D*—H	H⋯*A*	*D*⋯*A*	*D*—H⋯*A*
N1—H11*N*⋯Cl4^i^	0.90 (2)	2.38 (2)	3.218 (3)	155 (4)
N1—H12*N*⋯Cl2^ii^	0.90 (2)	2.36 (2)	3.241 (3)	166 (4)
N1—H13*N*⋯Cl1^iii^	0.88 (2)	2.29 (2)	3.143 (3)	165 (4)
O2—H2*O*⋯Cl3	0.82 (2)	2.28 (2)	3.092 (2)	170 (4)
N3—H31*N*⋯Cl1	0.88 (2)	2.34 (2)	3.208 (3)	171 (4)
N3—H32*N*⋯Cl2^iv^	0.90 (2)	2.39 (2)	3.289 (3)	176 (4)
N3—H33*N*⋯O11	0.90 (2)	2.35 (3)	3.072 (3)	137 (3)
N3—H33*N*⋯Cl2	0.90 (2)	2.74 (3)	3.361 (3)	127 (3)
N5—H51*N*⋯Cl1^v^	0.89 (2)	2.39 (3)	3.184 (3)	148 (4)
N5—H52*N*⋯Cl2^iii^	0.91 (2)	2.26 (2)	3.171 (3)	172 (4)
N5—H53*N*⋯Cl1^i^	0.88 (2)	2.32 (2)	3.194 (3)	171 (4)

Symmetry codes: (i) 
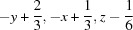
; (ii) 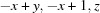; (iii) 
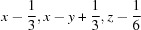
; (iv) 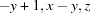; (v) 
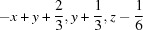
.

**Fig. 1.**
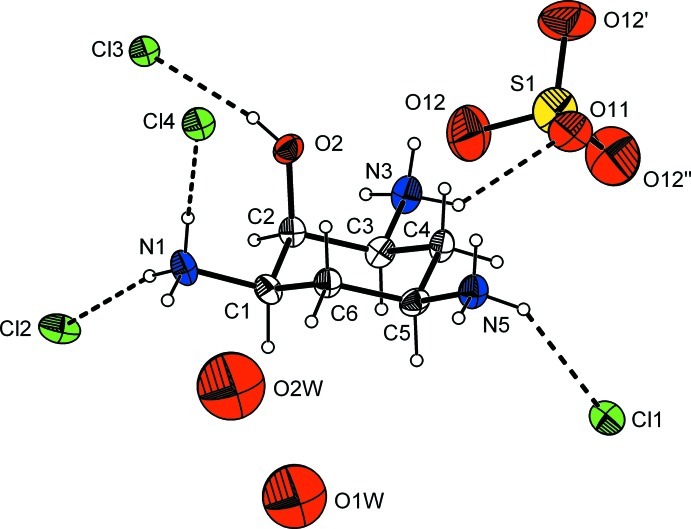
Ellipsoid plot (50% probability level) and numbering scheme of the title compound. Symmetry Codes: O12' 1 - y, x-y, z; O12" 1 - x + y, 1 - x, z.

**Fig. 2.**
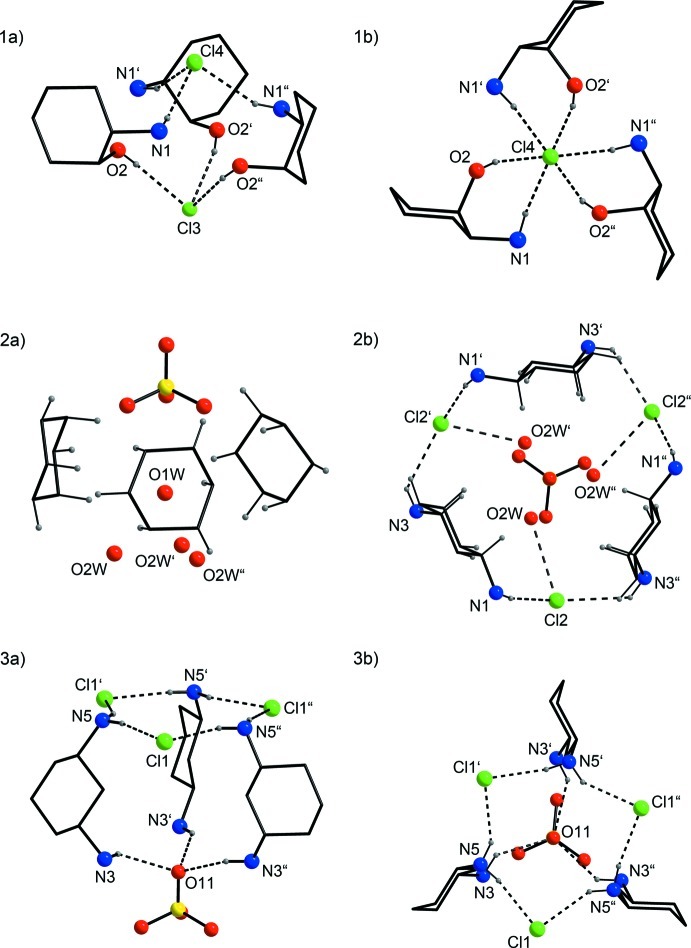
The three structural motives which are arranged along the threefold axis. (1) The [(H3L)3Cl2]7+ cage, (2) the lipophilic void generated by the C6H6-sides of three H3L3+ cations together with the disordered moieties which are found within this void, (3) the [(HSO4)(H3L)3Cl3]5+ cage. All substituents of the H3L3+ cation which are not essential have been omitted for clarity. (b) shows views along the threefold axis, (a) displays views for a perpendicular orientation. In 2b) the three chloride anions which keep the three cations together are also shown. They are omitted in 2a) for clarity.

## supplementary crystallographic information

### Comment

All-*cis*-2-hydroxycyclohexane-1,3,5-triamine (= *L*) has been prepared very recently in our laboratory for the first time by hydrogenation of picric acid (Merten *et al.*, 2012). Due to its two distinct, facially coordinating metal binding sites (*N*,*N*,*N**versus**N*,*O*,*N*), it is an interesting chelating agent. A corresponding diastereomer with the hydroxy group in *trans*-position has been known for many years (Castellanos *et al.*, 1980).

In the crystal structure of the title compound, the cyclohexane ring of the H_3_*L*^3+^ cation exhibits a chair conformation with the hydroxy group in axial and the three ammonium groups in equatorial position. Puckering parameters of the cyclohexane ring according to Cremer and Pople (1975) are Q = 0.588 Å, θ = 179.2 °, φ = 182.4 °. Due to the particular all-*cis*-configuration, the cation has an amphiphilic shape with a lipophilic (C_6_H_6_) and a hydrophilic (OH, NH_3_^+^) side. It is noteworthy that the lipophilic side of H_3_*L*^3+^ is directly oriented towards the *C*_3_-axis, generating thus a lipophilic void with a trigonal geometry. This void is filled with a part of the hydrogen sulfate anion and the water of crystallization, both located either on, or close to, the threefold axis. The moieties within this void are all disordered (see the experimental refinement section). The crystal structure is basically made up by a complex net of N—H···Cl hydrogen bonds. Additionally, the oxo oxygen atom O11 of the HSO_4_^-^ anion accepts three H(—N) hydrogen atoms, and the hydroxy group (O2) of the H_3_*L*^3+^ cation donates its proton to Cl3. O2 does, however, not act as an acceptor. A similar behaviour has recently been noted in related structures (Neis, Merten & Hegetschweiler, 2012; Neis, Merten & Altenhofer *et al.*, 2012). It is well known that the ability of axial hydroxy groups for forming hydrogen bonds is restricted on steric grounds (Bonnet *et al.*, 2005). Cl1 has a coordination number of four with a distorted tetrahedral geometry. Cl2 also accepts four H(—N) hydrogen atoms. However, if the Cl2···O2W distance of 3.225 Å is interpreted in terms of an O—H···Cl hydrogen bond, the coordination number is five with a geometry intermediate between a trigonal bipyramid and a square pyramid (τ = 0.43). It must, however, be emphasized that O2W is only partially occupied and the hydrogen atom in consideration could not be located (see again the experimental refinement section). Cl3 and Cl4 (lying on the *C*_3_-axis) have both a coordination number of three with a trigonal pyramidal geometry.

Viewing the structure along the threefold axis, three distinct structural motives can be recognized. (i) Cl3 and Cl4 together with three symmetry equivalent H_3_*L*^3+^ cations constitute a [(H_3_*L*)_3_Cl_2_]^7+^ cage, where Cl3 is hydrogen bonded to three hydroxy groups and Cl4 is hydrogen bonded to three ammonium groups of the three cations. (ii) The lipophilic void, formed by the C_6_H_6_-sides of three H_3_*L*^3+^ cations has already been mentioned. The three cations are interlinked by three Cl2 ions *via* N—H···Cl···H—N hydrogen bonding. The disorder that is observed for the moieties within this void, is probably caused by the absence of suitable hydrophilic hydrogen acceptors. (iii) Three H_3_*L*^3+^ cations together with a HSO_4_^-^ and three Cl^-^ counter ions form a [(H_3_*L*)_3_Cl_3_(HSO_4_)]^5+^ cage with the Cl^-^ anions and three ammonium groups (N5) forming an almost planar, hydrogen bonded N_3_H_6_Cl_3_ ring. Looking along the *C*_3_-axis, these three motives are arranged in the order cage 1 ··· lipophilic void ··· cage 2 ···.

### Experimental

A hydrated sulfate salt (H_3_*L*)_2_(SO_4_)_3_^.^5H_2_O has been prepared following the protocol given by Merten *et al.* (2012). ^1^H-NMR (D_2_O): δ (p.p.m.) = 1.92 (q, 2H), 2.25 (td, 2H), 3.51 (tt, 1H), 3.59 (ddd, 2H) 4.32 (t, 1H). ^13^C-NMR (D_2_O): δ (p.p.m.) = 29.7, 47.8, 52.0, 66.5. Elemental analysis calculated for C_12_H_46_N_6_O_19_S_3_ (%): C 21.36, H 6.87, N 12.46; found (%): C 21.47, H 6.13, N 12.07. Single crystals were obtained from an aqueous solution of the sulfate salt which has been acidified with conc. hydrochloric acid to pH < 1. The solution was allowed to evaporate slowly at ambient conditions (295 K). Single crystals appeared after a period of several days.

### Refinement

The H_3_*L*^3+^ cation could be refined without problems, and its hydrogen atoms could all be located. They were treated as recommended by Müller *et al.* (2006): A riding model was used for H(—C) atoms. The positional parameters of the O- and N-bonded H-atoms were refined using isotropic displacement parameters which were set to 1.5×*U*_eq_ of the pivot atom. In addition, restraints of 0.84 and 0.88 Å were used for the O—H and N—H distances. A total of four chloride positions were located; two of them (Cl3 and Cl4) are placed on the threefold axis, adding up altogether to a total charge of -2.667. Moreover, an SO_4_ moiety was located on the three fold axis. Although a hydrogen atom could not be found in proximity to any of the sulfate oxygen atoms, charge balance considerations require that this moiety must be formulated as HSO_4_^-^ (this is reasonable, if the acidic medium used for crystal growth is considered). In agreement with such an interpretation, two distinctly different S—O bond lengths were observed. O11, lying again on a threefold axis, forms a short S=O bond. O12, which forms a longer S—O bond, lies, however, on a general position. It appears thus that the hydrogen atom of the HSO_4_^-^ ion is distributed over three symmetry equivalent sites, and O12 is occupied in a 33%: 66% ratio by a hydroxy and an oxo group, respectively. Such a disorder is also reflected by the relatively large displacement of O12. In proximity to the disordered hydrogen sulfate anion, two additional peaks, O1W and O2W, were localized and were interpreted as disordered water molecules. O1W was again located on the threefold axis, whereas O2W lies on a general position. The short O1W···O2W interatomic distance of 2.46 Å precludes a simultaneous occupation of both positions. The occupancies of O1W and O2W were therefore constrained to add up to a value of 100%. The refinement exhibited equal distribution of 50% each, indicating that either one water molecule on O1W or three water molecules on O2W are present, resulting in a H_3_*L*^3+^: H_2_O ratio of 3:2. Due to this disorder, it was again not possible to locate any hydrogen atoms, and the relatively large displacement of O1W and O2W was refined isotropically. The Flack parameter (1255 Friedel pairs) refined to a value of 0.41 (7), indicating formation of an inversion-twin with roughly equal portions of the two domains. As a consequence, the TWIN option of *SHELXL* was used in the final refinement resulting in a marginal drop of wR2 from 10.3 to 10.1%. In agreement with the Flack parameter, the BASF parameter was found to be 41%.

### Crystal data

**Table:**

3C_6_H_18_N_3_O^3+^·8Cl^−^·HSO_4_^−^·2H_2_O	*D*_x_ = 1.419 Mg m^−^^3^
*M**_r_* = 861.40	Mo *K*α radiation, λ = 0.71073 Å
Trigonal, *R*3*c*	Cell parameters from 5824 reflections
*a* = 12.6549 (18) Å	θ = 3.3–38.0°
*c* = 43.616 (9) Å	µ = 0.66 mm^−^^1^
*V* = 6049.2 (17) Å^3^	*T* = 200 K
*Z* = 6	Prism, colourless
*F*(000) = 2724	0.48 × 0.40 × 0.32 mm

### Data collection

**Table:**

Stoe IPDS image plate diffractometer	2442 reflections with *I* > 2σ(*I*)
Radiation source: fine-focus sealed tube	*R*_int_ = 0.075
Graphite monochromator	θ_max_ = 25.5°, θ_min_ = 2.6°
phi scans	*h* = −14→15
14259 measured reflections	*k* = −15→15
2518 independent reflections	*l* = −52→52

### Refinement

**Table:**

Refinement on *F*^2^	Secondary atom site location: difference Fourier map
Least-squares matrix: full	Hydrogen site location: inferred from neighbouring sites
*R*[*F*^2^ > 2σ(*F*^2^)] = 0.037	H atoms treated by a mixture of independent and constrained refinement
*wR*(*F*^2^) = 0.101	*w* = 1/[σ^2^(*F*_o_^2^) + (0.0782*P*)^2^ + 1.7412*P*] where *P* = (*F*_o_^2^ + 2*F*_c_^2^)/3
*S* = 1.07	(Δ/σ)_max_ = 0.001
2518 reflections	Δρ_max_ = 0.75 e Å^−^^3^
169 parameters	Δρ_min_ = −0.32 e Å^−^^3^
11 restraints	Absolute structure: Flack (1983), 1255 Friedel pairs
Primary atom site location: structure-invariant direct methods	Flack parameter: 0.41 (7)

### Special details

**Table:**

Geometry. All e.s.d.'s (except the e.s.d. in the dihedral angle between two l.s. planes) are estimated using the full covariance matrix. The cell e.s.d.'s are taken into account individually in the estimation of e.s.d.'s in distances, angles and torsion angles; correlations between e.s.d.'s in cell parameters are only used when they are defined by crystal symmetry. An approximate (isotropic) treatment of cell e.s.d.'s is used for estimating e.s.d.'s involving l.s. planes.
Refinement. Refinement of *F*^2^ against ALL reflections. The weighted *R*-factor *wR* and goodness of fit *S* are based on *F*^2^, conventional *R*-factors *R* are based on *F*, with *F* set to zero for negative *F*^2^. The threshold expression of *F*^2^ > σ(*F*^2^) is used only for calculating *R*-factors(gt) *etc*. and is not relevant to the choice of reflections for refinement. *R*-factors based on *F*^2^ are statistically about twice as large as those based on *F*, and *R*- factors based on ALL data will be even larger.

### Fractional atomic coordinates and isotropic or equivalent isotropic displacement parameters (Å^2^)

**Table:**

	*x*	*y*	*z*	*U*_iso_*/*U*_eq_	Occ. (<1)
Cl1	0.25081 (6)	0.28514 (6)	0.523148 (15)	0.02924 (18)	
C1	0.2004 (2)	0.3248 (2)	0.40899 (6)	0.0217 (5)	
H1	0.2232	0.4124	0.4110	0.026*	
N1	0.0651 (2)	0.2485 (2)	0.40665 (6)	0.0260 (5)	
H11N	0.049 (4)	0.172 (2)	0.4029 (10)	0.039*	
H12N	0.033 (3)	0.264 (4)	0.4234 (7)	0.039*	
H13N	0.037 (4)	0.269 (4)	0.3907 (7)	0.039*	
C2	0.2434 (2)	0.2867 (2)	0.43791 (6)	0.0201 (5)	
H2	0.2079	0.3027	0.4566	0.024*	
O2	0.20562 (18)	0.16022 (19)	0.43620 (4)	0.0257 (4)	
H2O	0.153 (3)	0.111 (3)	0.4479 (8)	0.039*	
C3	0.3833 (2)	0.3621 (3)	0.43928 (6)	0.0221 (5)	
H3	0.4084	0.4501	0.4418	0.027*	
N3	0.4276 (2)	0.3228 (3)	0.46641 (5)	0.0272 (5)	
H31N	0.382 (3)	0.321 (4)	0.4818 (7)	0.041*	
H32N	0.418 (4)	0.248 (2)	0.4636 (9)	0.041*	
H33N	0.5097 (18)	0.365 (3)	0.4673 (10)	0.041*	
C4	0.4447 (2)	0.3482 (3)	0.41051 (6)	0.0232 (5)	
H4A	0.5343	0.4014	0.4121	0.028*	
H4B	0.4262	0.2627	0.4086	0.028*	
C5	0.3974 (3)	0.3838 (2)	0.38230 (6)	0.0224 (5)	
H5	0.4231	0.4723	0.3836	0.027*	
N5	0.4537 (2)	0.3630 (2)	0.35426 (5)	0.0255 (5)	
H51N	0.5342 (18)	0.413 (3)	0.3532 (9)	0.038*	
H52N	0.424 (4)	0.373 (4)	0.3361 (6)	0.038*	
H53N	0.438 (4)	0.287 (2)	0.3530 (9)	0.038*	
C6	0.2583 (2)	0.3090 (3)	0.37982 (6)	0.0214 (5)	
H6A	0.2323	0.2218	0.3769	0.026*	
H6B	0.2308	0.3365	0.3618	0.026*	
S1	0.6667	0.3333	0.51658 (4)	0.0494 (4)	
O11	0.6667	0.3333	0.48521 (12)	0.0487 (11)	
O12	0.5574 (4)	0.3375 (4)	0.52786 (9)	0.0796 (11)	
Cl2	0.65819 (7)	0.61189 (6)	0.460229 (16)	0.03246 (19)	
Cl3	0.0000	0.0000	0.48180 (3)	0.0243 (3)	
Cl4	0.3333	0.6667	0.53799 (3)	0.0309 (3)	
O1W	0.3333	0.6667	0.4283 (5)	0.113 (6)*	0.502 (13)
O2W	0.2408 (13)	0.5251 (14)	0.4717 (3)	0.127 (5)*	0.498 (13)

### Atomic displacement parameters (Å^2^)

**Table:**

	*U*^11^	*U*^22^	*U*^33^	*U*^12^	*U*^13^	*U*^23^
Cl1	0.0288 (3)	0.0305 (3)	0.0258 (3)	0.0128 (3)	−0.0011 (3)	−0.0080 (3)
C1	0.0228 (13)	0.0266 (13)	0.0200 (12)	0.0155 (11)	−0.0017 (10)	−0.0011 (10)
N1	0.0236 (12)	0.0382 (13)	0.0228 (12)	0.0205 (11)	0.0001 (10)	0.0006 (10)
C2	0.0195 (12)	0.0250 (13)	0.0157 (12)	0.0111 (11)	−0.0007 (9)	−0.0012 (9)
O2	0.0232 (10)	0.0249 (10)	0.0256 (10)	0.0094 (8)	0.0020 (8)	0.0070 (8)
C3	0.0224 (13)	0.0241 (13)	0.0164 (12)	0.0090 (11)	−0.0022 (10)	−0.0020 (10)
N3	0.0227 (12)	0.0378 (14)	0.0165 (12)	0.0117 (11)	−0.0040 (9)	0.0008 (10)
C4	0.0165 (12)	0.0293 (14)	0.0211 (14)	0.0096 (12)	0.0013 (10)	0.0035 (11)
C5	0.0253 (13)	0.0216 (12)	0.0184 (12)	0.0103 (11)	0.0051 (10)	0.0041 (10)
N5	0.0250 (12)	0.0334 (13)	0.0171 (11)	0.0139 (10)	0.0029 (9)	0.0042 (10)
C6	0.0213 (12)	0.0262 (13)	0.0167 (12)	0.0119 (11)	0.0011 (9)	0.0039 (10)
S1	0.0587 (6)	0.0587 (6)	0.0308 (7)	0.0294 (3)	0.000	0.000
O11	0.0493 (17)	0.0493 (17)	0.047 (3)	0.0246 (8)	0.000	0.000
O12	0.073 (2)	0.085 (3)	0.090 (2)	0.046 (2)	0.041 (2)	0.003 (2)
Cl2	0.0304 (4)	0.0296 (3)	0.0231 (3)	0.0044 (3)	−0.0005 (3)	0.0005 (3)
Cl3	0.0252 (3)	0.0252 (3)	0.0225 (5)	0.01259 (17)	0.000	0.000
Cl4	0.0292 (4)	0.0292 (4)	0.0343 (6)	0.01459 (19)	0.000	0.000

### Geometric parameters (Å, º)

**Table:**

C1—N1	1.490 (3)	N3—H33N	0.901 (19)
C1—C6	1.530 (4)	C4—C5	1.531 (4)
C1—C2	1.543 (4)	C4—H4A	0.9900
C1—H1	1.0000	C4—H4B	0.9900
N1—H11N	0.896 (19)	C5—N5	1.504 (4)
N1—H12N	0.904 (19)	C5—C6	1.529 (4)
N1—H13N	0.875 (19)	C5—H5	1.0000
C2—O2	1.425 (3)	N5—H51N	0.893 (19)
C2—C3	1.536 (4)	N5—H52N	0.912 (19)
C2—H2	1.0000	N5—H53N	0.881 (19)
O2—H2O	0.823 (19)	C6—H6A	0.9900
C3—N3	1.497 (3)	C6—H6B	0.9900
C3—C4	1.531 (4)	S1—O11	1.368 (5)
C3—H3	1.0000	S1—O12^i^	1.493 (3)
N3—H31N	0.876 (19)	S1—O12^ii^	1.493 (3)
N3—H32N	0.901 (19)	S1—O12	1.493 (3)
			
N1—C1—C6	109.2 (2)	C5—C4—C3	109.2 (2)
N1—C1—C2	108.9 (2)	C5—C4—H4A	109.8
C6—C1—C2	111.8 (2)	C3—C4—H4A	109.8
N1—C1—H1	108.9	C5—C4—H4B	109.8
C6—C1—H1	108.9	C3—C4—H4B	109.8
C2—C1—H1	108.9	H4A—C4—H4B	108.3
C1—N1—H11N	106 (3)	N5—C5—C6	109.5 (2)
C1—N1—H12N	108 (3)	N5—C5—C4	108.2 (2)
H11N—N1—H12N	119 (4)	C6—C5—C4	111.9 (2)
C1—N1—H13N	112 (3)	N5—C5—H5	109.1
H11N—N1—H13N	105 (4)	C6—C5—H5	109.1
H12N—N1—H13N	107 (4)	C4—C5—H5	109.1
O2—C2—C3	109.6 (2)	C5—N5—H51N	113 (3)
O2—C2—C1	109.6 (2)	C5—N5—H52N	114 (3)
C3—C2—C1	108.3 (2)	H51N—N5—H52N	105 (4)
O2—C2—H2	109.8	C5—N5—H53N	112 (3)
C3—C2—H2	109.8	H51N—N5—H53N	109 (4)
C1—C2—H2	109.8	H52N—N5—H53N	103 (4)
C2—O2—H2O	121 (3)	C5—C6—C1	109.8 (2)
N3—C3—C4	108.3 (2)	C5—C6—H6A	109.7
N3—C3—C2	109.3 (2)	C1—C6—H6A	109.7
C4—C3—C2	112.9 (2)	C5—C6—H6B	109.7
N3—C3—H3	108.7	C1—C6—H6B	109.7
C4—C3—H3	108.7	H6A—C6—H6B	108.2
C2—C3—H3	108.7	O11—S1—O12^i^	109.24 (18)
C3—N3—H31N	105 (3)	O11—S1—O12^ii^	109.24 (19)
C3—N3—H32N	111 (3)	O12^i^—S1—O12^ii^	109.70 (18)
H31N—N3—H32N	110 (4)	O11—S1—O12	109.24 (18)
C3—N3—H33N	110 (3)	O12^i^—S1—O12	109.70 (18)
H31N—N3—H33N	122 (4)	O12^ii^—S1—O12	109.70 (18)
H32N—N3—H33N	98 (4)		
			
N1—C1—C2—O2	58.1 (3)	N3—C3—C4—C5	178.1 (2)
C6—C1—C2—O2	−62.7 (3)	C2—C3—C4—C5	56.8 (3)
N1—C1—C2—C3	177.6 (2)	C3—C4—C5—N5	−177.0 (2)
C6—C1—C2—C3	56.8 (3)	C3—C4—C5—C6	−56.3 (3)
O2—C2—C3—N3	−58.0 (3)	N5—C5—C6—C1	177.2 (2)
C1—C2—C3—N3	−177.5 (2)	C4—C5—C6—C1	57.2 (3)
O2—C2—C3—C4	62.7 (3)	N1—C1—C6—C5	−178.3 (2)
C1—C2—C3—C4	−56.8 (3)	C2—C1—C6—C5	−57.7 (3)

Symmetry codes: (i) −*y*+1, *x*−*y*, *z*; (ii) −*x*+*y*+1, −*x*+1, *z*.

### Hydrogen-bond geometry (Å, º)

**Table:**

*D*—H···*A*	*D*—H	H···*A*	*D*···*A*	*D*—H···*A*
N1—H11*N*···Cl4^iii^	0.90 (2)	2.38 (2)	3.218 (3)	155 (4)
N1—H12*N*···Cl2^iv^	0.90 (2)	2.36 (2)	3.241 (3)	166 (4)
N1—H13*N*···Cl1^v^	0.88 (2)	2.29 (2)	3.143 (3)	165 (4)
O2—H2*O*···Cl3	0.82 (2)	2.28 (2)	3.092 (2)	170 (4)
N3—H31*N*···Cl1	0.88 (2)	2.34 (2)	3.208 (3)	171 (4)
N3—H32*N*···Cl2^i^	0.90 (2)	2.39 (2)	3.289 (3)	176 (4)
N3—H33*N*···O11	0.90 (2)	2.35 (3)	3.072 (3)	137 (3)
N3—H33*N*···Cl2	0.90 (2)	2.74 (3)	3.361 (3)	127 (3)
N5—H51*N*···Cl1^vi^	0.89 (2)	2.39 (3)	3.184 (3)	148 (4)
N5—H52*N*···Cl2^v^	0.91 (2)	2.26 (2)	3.171 (3)	172 (4)
N5—H53*N*···Cl1^iii^	0.88 (2)	2.32 (2)	3.194 (3)	171 (4)

Symmetry codes: (i) −*y*+1, *x*−*y*, *z*; (iii) −*y*+2/3, −*x*+1/3, *z*−1/6; (iv) −*x*+*y*, −*x*+1, *z*; (v) *x*−1/3, *x*−*y*+1/3, *z*−1/6; (vi) −*x*+*y*+2/3, *y*+1/3, *z*−1/6.
